# AI improves consistency in regional brain volumes measured in ultra-low-field MRI and 3T MRI

**DOI:** 10.3389/fnimg.2025.1588487

**Published:** 2025-06-04

**Authors:** Kh Tohidul Islam, Shenjun Zhong, Parisa Zakavi, Helen Kavnoudias, Shawna Farquharson, Gail Durbridge, Markus Barth, Andrew Dwyer, Katie L. McMahon, Paul M. Parizel, Richard McIntyre, Gary F. Egan, Meng Law, Zhaolin Chen

**Affiliations:** ^1^Monash Biomedical Imaging, Monash University, Clayton, VIC, Australia; ^2^Department of Radiology, The Alfred, Melbourne, VIC, Australia; ^3^Department of Surgery, School of Translational Medicine, Monash University, Clayton, VIC, Australia; ^4^Australian National Imaging Facility, Brisbane, QLD, Australia; ^5^Herston Imaging Research Facility, University of Queensland, Brisbane, QLD, Australia; ^6^School of Electrical Engineering and Computer Science, University of Queensland, Brisbane, QLD, Australia; ^7^Centre for Advanced Imaging, Australian Institute for Bioengineering and Nanotechnology, University of Queensland, Brisbane, QLD, Australia; ^8^South Australian Health and Medical Research Institute, Adelaide, SA, Australia; ^9^SA Medical Imaging, SA Health, Adelaide, SA, Australia; ^10^School of Clinical Sciences, Faculty of Health, Queensland University of Technology, Brisbane, QLD, Australia; ^11^David Hartley Chair of Radiology, Royal Perth Hospital, Perth, WA, Australia; ^12^Medical School, University of Western Australia, Perth, WA, Australia; ^13^Monash Healthcare Network, Melbourne, VIC, Australia; ^14^Department of Neuroscience, School of Translational Medicine, Monash University, Clayton, VIC, Australia; ^15^Department of Data Science and AI, Monash University, Clayton, VIC, Australia

**Keywords:** accessible MRI, ultra-low-field MRI, deep learning in neuroimaging, brain volume measurement, quantitative MRI analysis

## Abstract

This study compares volumetric measurements of various brain regions using different magnetic resonance imaging (MRI) modalities and deep learning models, specifically 3T MRI, ultra-low field (ULF) MRI at 64mT, and AI-enhanced ULF MRI using SynthSR and HiLoResGAN. The aim is to evaluate the alignment and agreement among field strengths and ULF MRI with and without AI. Descriptive statistics, paired *t*-tests, effect size analyses, and regression analyses are employed to assess the relationships and differences between modalities. The results indicate that volumetric measurements derived from 64mT MRI deviate significantly from those obtained using 3T MRI. By leveraging SynthSR and LoHiResGAN models, these deviations are reduced, bringing the volumetric estimates closer to those obtained from 3T MRI, which serves as the reference standard for brain volume quantification. These findings highlight that deep learning models can reduce systematic differences in brain volume measurements across field strengths, providing potential solutions to minimize bias in imaging studies.

## 1 Introduction

Magnetic resonance imaging (MRI) is a non-invasive medical imaging modality renowned for its exceptional soft-tissue contrast, making it indispensable for brain imaging and neurodiagnostic applications (Du et al., 2024). However, high-field MRI scanners (1.5T–3.0T), while offering superior resolution and signal-to-noise ratio (SNR), are expensive and less accessible, particularly in resource-limited and remote settings (Dietzel et al., 2024). Beyond cost and infrastructure challenges, high-field MRI systems face operational limitations (Kimberly et al., 2023). They are immobile, requiring patients to be transported to specialized imaging facilities, which may not be feasible in emergency or bedside scenarios (Shoghli et al., 2023). Safety concerns, including the risks of thermal heating, acoustic noise, and interactions with ferromagnetic materials, necessitate rigorous protocols and patient monitoring (Yuen et al., 2022). Furthermore, the confined space of high-field scanners can cause discomfort or claustrophobia, limiting patient compliance (Lawal et al., 2024). This limitation has driven interest in ultra-low-field MRI (ULF-MRI) systems, such as the 64mT Hyperfine Swoop scanner (Shen et al., 2024), which provide a cost-effective, portable, and scalable alternative (Altaf et al., 2023; Morey et al., 2025). Despite their advantages, ULF-MRI scanners suffer from lower image quality and reduced resolution (Lau et al., 2023; Altaf et al., 2024; Dayarathna et al., 2024a), which can hinder accurate quantitative measurements, such as brain volumes, critical for neurological diagnosis and monitoring (Arnold et al., 2022; Abate et al., 2024; Okar et al., 2025).

Given the crucial role of neuroimaging in diagnosing and monitoring neurological conditions, such as Alzheimer's disease (Sorby-Adams et al., 2024; Mathew et al., 2023), multiple sclerosis (Fujimori and Nakashima, 2024), seizure (Bauer et al., 2025), and traumatic brain injury (Wells et al., 2024), accurate volumetric measurements of brain regions are essential (Aman et al., 2025; Seehafer et al., 2025). These measurements provide valuable insights into disease progression, support treatment planning, and enable the evaluation of therapeutic outcomes (Schweizer et al., 2023). While high-field MRI systems remain the standard for acquiring reliable volumetric data due to their superior resolution and SNR (Salvolini and Scarabino, 2006), their cost and accessibility challenges necessitate the exploration of alternative methods. Ultra-low-field MRI (ULF-MRI) has emerged as a promising option to address these challenges, offering potential solutions for obtaining reliable volumetric measurements in resource-limited settings (Iglesias et al., 2023b; DesRoche et al., 2024).

ULF-MRI offers several advantages, including lower costs, reduced power consumption, and portability, making it suitable for bedside imaging and resource-constrained environments (DesRoche et al., 2024; Khanduja et al., 2025). Sheth et al. (2021) demonstrated the effectiveness of ULF-MRI in assessing brain injuries in intensive care units and remote settings, enabling patient diagnosis without requiring transport. Similarly, Kimberly et al. (2023) highlighted the benefits of ULF-MRI for rapid diagnostic imaging in emergency and intensive care settings, reducing disparities in access to neuroimaging.

Despite these advantages, a significant limitation of ULF-MRI is its lower signal-to-noise ratio (SNR), resulting in poorer image quality and reduced resolution compared to high-field MRI (Ayde et al., 2024). Recent advancements in super-resolution techniques have shown promise in addressing these limitations (Baljer et al., 2024; Laso et al., 2023; Kuoy et al., 2022; Lau et al., 2023; Cooper et al., 2024; Dayarathna et al., 2024b). For instance, Baljer et al. (2024) proposed a deep learning-based super-resolution framework leveraging multi-orientation U-Net to reconstruct high-resolution isotropic T2-weighted scans from low-resolution pediatric ULF-MRI. Their approach significantly improved the volumetric accuracy of deep brain structures, achieving high correlation with high-field MRI. Similarly, Laso et al. (2023) demonstrated that super-resolution methods can effectively quantify brain volumes and white matter hyperintensity, achieving strong correlations with high-field MRI. Kuoy et al. (2022) further emphasized the utility of point-of-care ULF-MRI for bedside imaging, while Lau et al. (2023) demonstrated that multi-orientation image averaging combined with machine learning significantly improves SNR and image resolution.

To overcome the limitations of ULF-MRI, recent advancements in deep learning have facilitated the development of models such as SynthSR (Iglesias et al., 2023a) and LoHiResGAN (Islam et al., 2023). SynthSR, a convolutional neural network (CNN)-based model, processes both T1- and T2-weighted images to generate high-resolution synthetic MRI images. LoHiResGAN, which signifies the low-field to high-field translation task, employing a generative adversarial network (GAN) architecture with ResNet components, enhances the quality and resolution of ULF-MRI images to levels comparable with high-field MRI scans. These deep learning models contribute to better alignment and consistency of ULF-MRI volumetric measurements with those from the 3T reference, thereby narrowing the disparities between ultra-low-field and high-field imaging.

This study aims to evaluate the effectiveness of SynthSR and LoHiResGAN in translating ULF-MRI (64mT) images into higher fidelity representations that yield volumetric measurements closely aligned with those obtained from 3T MRI, the widely accepted reference standard for neuroimaging (Seiger et al., 2015). By comparing volumetric measurements across 19 distinct brain regions from 3T MRI, 64mT MRI, SynthSR, and LoHiResGAN-generated images, we seek to assess the accuracy and reliability of these models. Our goal is to explore the potential of these advanced models to enhance the compatibility of ULF-MRI with high-field MRI standards, thereby expanding the accessibility and utility of MRI technology in various clinical and research settings.

## 2 Materials and methods

### 2.1 Data collection and deep learning models

Institutional ethics and institutional review board (IRB) approvals were obtained from the Monash University Human Research Ethics Committee, and written informed consent was acquired from all participants involved in the study. All experiments adhered to relevant guidelines and regulations, with consideration given to the Good Machine Learning Practices (GMLPs) checklist where applicable (Aggarwal et al., 2023). The study, conducted between October 2022 and June 2023, involved 92 healthy individuals (mean age 44; range 18–81; SD = 17, 42 males). Among them, 50 was used to test the model, while the LoHiResGAN model was trained in our previous study using the remaining data from the same cohort (Islam et al., 2023). Each participant was scanned at Monash Biomedical Imaging using both Hyperfine Swoop (64mT) and Siemens Biograph mMR (3T) imaging systems.

For the 64mT Hyperfine Swoop system, T1-weighted and T2-weighted MRI sequences were acquired to generate the required data for subsequent processing with the SynthSR model. The scanning parameters for the 64mT system included a voxel resolution of 1.60 × 1.60 × 5.00mm^3^ for T1-weighted images and 1.50 × 1.50 × 5.00mm^3^ for T2-weighted images. For the 3T Siemens Biograph mMR system, T1-weighted MP-RAGE images with a voxel resolution of 1.00 × 1.00 × 1.00mm^3^ were acquired.

The collected data underwent separate preprocessing pipelines for the LoHiResGAN and SynthSR models, with a shared initial step. Specifically, for both models, we first applied bias field correction to the 64mT scans using FSL-FAST, without referencing an external atlas for spatial information (Zhang et al., 2001). For LoHiResGAN, following bias correction, the T1-weighted images from the 64mT scans were resampled to 1.00 mm isotropic resolution. We then performed co-registration between the resampled 64mT and the 3T T1-weighted images using FMRIB's linear image registration tool (FLIRT) to ensure precise alignment, thereby producing paired datasets for downstream model application (Jenkinson et al., 2002). For SynthSR, after bias correction, the 64mT T1- and T2-weighted images were first co-registered using FLIRT to create a combined input dataset. This co-registered multi-contrast input was processed by the SynthSR model, which generated a high-resolution synthetic T1-weighted image at 1.00 mm isotropic resolution. Finally, the SynthSR-generated image was co-registered with the 3T T1-weighted image using FLIRT to enable a fair and consistent comparison in subsequent volumetric analyses.

The LoHiResGAN (Low-to-High-Resolution Generative Adversarial Network) model, previously developed and pre-trained on our paired 64mT and 3T dataset (Islam et al., 2023), was applied directly to the original 64mT MRI scans to enhance their image quality. Unlike SynthSR, which requires both T1- and T2-weighted images as inputs, LoHiResGAN operates solely on T1-weighted images from the 64mT system, utilizing a generative adversarial network (GAN) architecture to improve resolution and anatomical detail. SynthSR, on the other hand, is a publicly available pre-trained model designed for broad generalization across multiple scanners and contrasts, including ULF protocols. Given its extensive multi-institutional training and demonstrated compatibility with ULF data, we employed SynthSR in its original form without further retraining on our specific dataset. Both SynthSR and LoHiResGAN were applied independently as separate enhancement pathways to the raw ULF data, providing two distinct outputs for comparison. The enhanced images generated by these two models were subsequently compared with the original 3T T1-weighted images to evaluate the alignment and consistency of brain volumetric measurements across different methods.

**Figure 1:**
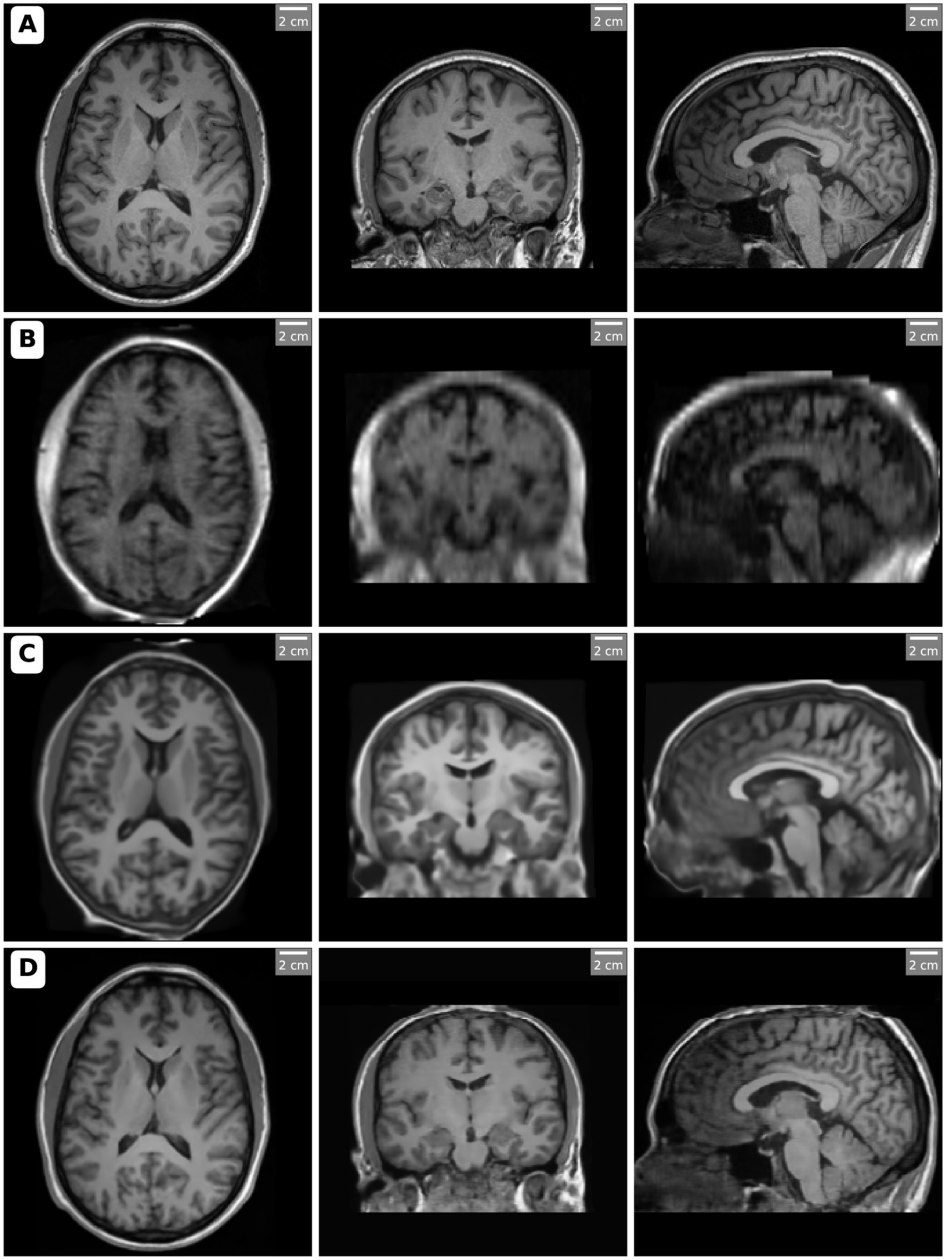
Orthogonal views (axial, coronal, and sagittal slices) of MRI scans acquired and processed with different methods. Each row corresponds to a specific imaging method: **(A)** High-field MRI (3T), **(B)** Ultra-low-field MRI (64mT), **(C)** SynthSR-enhanced MRI, and **(D)** LoHiResGAN-enhanced MRI. The slices highlight the differences in image quality and anatomical clarity, with SynthSR and LoHiResGAN demonstrating significant enhancements in resolution and contrast for ultra-low-field MRI scans. Notably, in the SynthSR-enhanced images, artificially increased gray matter contrast is observed in certain regions, such as the frontal lobes, likely due to the model's integration of T1 and T2 inputs and its aggressive contrast enhancement strategy.

In this study, we focused on T1-weighted images for volumetric comparison analysis due to their superior soft-tissue contrast and relevance in brain morphometry. The volumetric measurements obtained from the 3T MRI, 64mT MRI, SynthSR, and LoHiResGAN models were analyzed to evaluate the performance of these methods in replicating high-field MRI measurements. Figure 1 presents orthogonal views (axial, coronal, and sagittal slices) of MRI scans acquired using different methods, showcasing the variations in image quality and enhancements.

### 2.2 Brain segmentation and analysis

This study merged left and right hemisphere volumes to reduce inter-subject anatomical variability and focus on assessing overall volumetric agreement between methods rather than hemispheric asymmetries. This approach ensured a more robust comparison across modalities, given our sample size and study objectives. To produce the brain region masks, we utilized the SynthSeg^+^ segmentation tools, as detailed in the work by Billot et al. (2023). SynthSeg^+^ provides robust segmentation of heterogeneous clinical brain MRI datasets by leveraging deep learning models trained on synthetic data with domain randomization. This approach enables the segmentation of brain scans with varying contrasts and resolutions without requiring retraining. SynthSeg^+^ performs whole-brain segmentation, cortical parcellation, and intracranial volume estimation. Additionally, SynthSeg^+^ internally resamples all input images to 1.00 mm isotropic resolution prior to segmentation, ensuring standardized voxel dimensions and facilitating robust segmentation across modalities.

Initially, SynthSeg^+^ generates 33 individual brain region masks, which include separate masks for the left and right hemispheres. For the purposes of this study, these masks were merged to create 19 distinct brain regions. The following regions were included in the analysis: Total Intracranial, 3rd Ventricle, 4th Ventricle, Brain-Stem, cerebrospinal fluid CSF, Cerebral White Matter, Cerebral Cortex, Lateral Ventricle, Inferior Lateral Ventricle, Cerebellum White Matter, Cerebellum Cortex, Thalamus, Caudate, Putamen, Pallidum, Hippocampus, Amygdala, Accumbens Area, and Ventral DC.

The segmentation masks obtained from SynthSeg^+^ were then used to extract volumetric measurements from the 3T MRI, 64mT MRI, SynthSR, and LoHiResGAN datasets. For SynthSR data generation, both T1- and T2-weighted images from the 64mT MRI were jointly used as inputs to produce an enhanced T1-weighted image. In contrast, LoHiResGAN utilized only the T1-weighted images for enhancement. In our subsequent volumetric analysis, we focused on the T1-weighted outputs from both models to ensure consistency across methods. All brain region segmentations, including for the 3T MRI, were generated using SynthSeg^+^. While no manual corrections were applied, we performed qualitative visual checks to ensure reasonable segmentation accuracy.

### 2.3 Model architecture

The SynthSR model employs a super-resolution approach to generate high-resolution T1-weighted images from various MRI scans, leveraging a convolutional neural network (CNN) that synthesizes isotropic 1 mm images from clinical MRI data of varying orientations, resolutions, and contrasts. This technique enhances the accuracy of quantitative brain morphometry, even with ultra-low-field MRI data, by producing consistent and high-quality volumetric measurements across different brain regions.

The LoHiResGAN model uses a generative adversarial network (GAN) architecture incorporating ResNet components. This model translates ultra-low-field MRI images (64mT) to high-field MRI quality (3T) by enhancing the image quality, signal-to-noise ratio, and spatial resolution. The LoHiResGAN model consists of a generator and discriminator, where the generator utilizes ResNet-based downsampling and upsampling blocks to preserve fine-grained details and capture long-range dependencies in the images. This architecture significantly improves the translated images' structural similarity and perceptual quality, making them more comparable to high-field MRI scans.

Our study compares these models to leverage SynthSR's strengths in producing high-resolution images and LoHiResGAN's capabilities in enhancing ultra-low-field MRI data to high-field quality, thereby improving the overall accuracy and reliability of volumetric measurements in neuroimaging.

### 2.4 Statistical analysis and visualization

Descriptive statistics were calculated for volumetric measurements across all brain regions to summarize the data. Paired *t*-tests were conducted to compare the volumetric measurements between the 3T MRI dataset and the other datasets (64mT MRI, SynthSR, and LoHiResGAN). Effect sizes (Cohen's *d*) were also calculated to assess the magnitude of differences between the datasets.

Regression analysis evaluated the linear relationship between volumetric measurements from 3T MRI and the other datasets. The regression results were summarized, including coefficients, R-squared values, and *p*-values, to understand the predictive accuracy of the ultra-low-field MRI and deep learning models in replicating high-field MRI measurements.

Scatter plots were created to illustrate the correlation between volumetric measurements from 3T MRI and the other datasets for visualization. Bland-Altman plots were produced to assess the agreement between volumetric measurements from 3T MRI and the different datasets, highlighting any discrepancies and the limits of agreement. Additionally, correlation heatmaps were generated to provide a comprehensive view of the relationships between volumetric measurements within each dataset.

These analyses and visualizations help to explain the similarities and differences in volumetric measurements across the different MRI methods, providing a robust framework for comparing the alignment of ultra-low-field MRI and deep learning-based models with reference high-field MRI measurements.

## 3 Results

**Figure 2:**
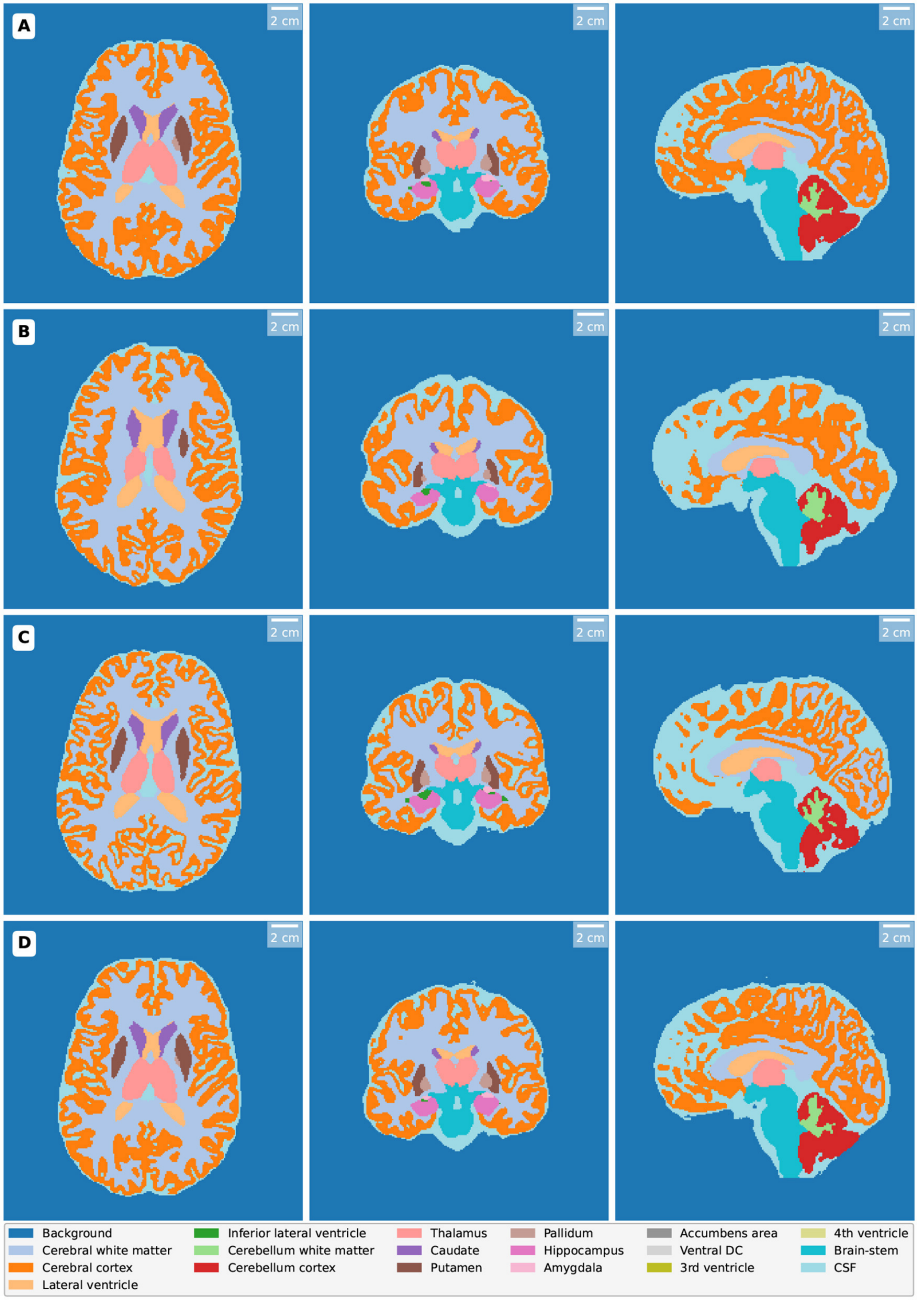
Segmentation results for orthogonal views (axial, coronal, and sagittal slices) from MRI scans processed using various methods. Each row represents a different imaging method: **(A)** High-field MRI (3T) demonstrates detailed segmentation with high accuracy and contrast. **(B)** Ultra-low-field MRI (64mT) exhibits reduced segmentation precision due to lower resolution and image quality. **(C)** SynthSR-enhanced MRI improves segmentation consistency through super-resolution techniques. **(D)** LoHiResGAN-enhanced MRI further enhances segmentation quality using deep learning-based image enhancement.

This section presents the findings of the study, comparing volumetric measurements across different MRI techniques and models, namely 3T MRI, 64mT MRI, SynthSR, and LoHiResGAN. The results are organized into several subsections, starting with descriptive statistics, followed by paired *t*-tests and effect sizes, regression analysis, scatter plot analysis, Bland-Altman plot analysis, and correlation heatmap analysis. Each subsection provides a detailed examination of the data, highlighting the agreement and alignment of the volumetric measurements obtained from each method with the reference high-field MRI. Figure 2 depicts segmentation results for orthogonal views (axial, coronal, and sagittal slices) of MRI scans processed using different modalities and enhancement methods.

### 3.1 Summary of volumetric measurements

The volumetric measurements across different MRI techniques and models (3T MRI, 64mT MRI, SynthSR, and LoHiResGAN) are presented in Table 1. These values represent the mean and standard deviation across subjects for each brain region. Overall, total intracranial volume remains relatively consistent across methods, with LoHiResGAN showing the closest match to 3T MRI (Δ = +0.89%), followed by SynthSR (Δ = +1.72%) and 64mT MRI (Δ = −0.74%), indicating stable measurements across imaging techniques.

**Table 1:** Summary of volumetric measurements across methods (mean ± SD) in 10^3^ mm^3^ with percentage differences (Δ) from 3T MRI.

**Brain region**	**3T MRI (Reference Std.)**	**64mT MRI**		**SynthSR**		**LoHiResGAN**	
		**Mean** ± **SD**	Δ **(%)**	**Mean** ± **SD**	Δ **(%)**	**Mean** ± **SD**	Δ **(%)**
Total intracranial	1, 531.25 ± 139.52	1519.91 ± 132.47	–0.74%	1557.52 ± 134.28	+1.72%	1544.83 ± 136.89	+0.89%
3rd ventricle	0.92 ± 0.31	1.16 ± 0.35^**^	+26.1%	1.18 ± 0.37^**^	+28.3%	0.90 ± 0.30	–2.2%
4th ventricle	1.68 ± 0.45	2.19 ± 0.62^**^	+30.4%	2.32 ± 0.53^**^	+38.1%	1.69 ± 0.45	+0.6%
Brain-stem	23.67 ± 2.32	22.57 ± 2.69	–4.6%	23.43 ± 2.11	–1.0%	23.70 ± 2.26	+0.1%
CSF	232.58 ± 32.77	287.73 ± 27.36^***^	+23.7%	271.16 ± 28.12^**^	+16.6%	246.64 ± 31.54	+6.0%
Cerebral white matter	482.03 ± 55.59	474.54 ± 50.04	–1.6%	480.70 ± 49.93	–0.3%	487.48 ± 55.35	+1.1%
Cerebral cortex	562.41 ± 53.60	522.71 ± 45.73^**^	–7.1%	550.39 ± 49.20	–2.1%	556.37 ± 51.73	–1.1%
Lateral ventricle	16.93 ± 8.78	26.06 ± 10.41^***^	+54.0%	26.21 ± 12.78^***^	+55.0%	16.89 ± 8.77	–0.2%
Inferior lateral ventricle	0.89 ± 0.20	0.44 ± 0.27^***^	–50.6%	1.58 ± 0.60^**^	+77.5%	0.85 ± 0.20	–4.5%
Cerebellum white matter	31.04 ± 3.73	32.32 ± 3.43	+4.1%	31.19 ± 3.45	+0.5%	32.03 ± 3.54	+3.2%
Cerebellum cortex	116.88 ± 12.12	98.78 ± 9.81^**^	–15.5%	108.12 ± 10.83	–7.5%	116.52 ± 11.79	–0.3%
Thalamus	15.34 ± 1.76	13.59 ± 1.64^**^	–11.4%	14.00 ± 1.84	–8.7%	15.28 ± 1.75	–0.4%
Caudate	8.42 ± 1.00	7.48 ± 0.79**	–11.2%	8.41 ± 0.96	–0.1%	8.31 ± 0.97	–1.3%
Putamen	11.60 ± 1.37	9.23 ± 1.25^***^	–20.4%	11.77 ± 1.49	+1.5%	11.45 ± 1.38	–1.3%
Pallidum	3.53 ± 0.43	2.18 ± 0.46^***^	–38.2%	3.80 ± 0.54	+7.6%	3.53 ± 0.43	0.0%
Hippocampus	8.60 ± 0.80	7.24 ± 0.89^***^	–15.8%	9.20 ± 1.12	+7.0%	8.47 ± 0.80	–1.5%
Amygdala	3.93 ± 0.45	3.05 ± 0.37^**^	–22.4%	3.76 ± 0.51	–4.3%	3.88 ± 0.44	–1.3%
Accumbens area	1.58 ± 0.21	1.09 ± 0.15^***^	–31.0%	1.47 ± 0.20	–7.0%	1.54 ± 0.19	–2.5%
Ventral DC	9.21 ± 0.92	7.56 ± 0.83^**^	–17.9%	8.82 ± 0.94	–4.2%	9.30 ± 0.94	+1.0%

Significance levels: *p* < 0.05 (^*^), *p* < 0.01 (^**^ ), *p* < 0.001 (^***^).

Notably, discrepancies emerge for fluid-filled compartments such as the ventricles and cerebrospinal fluid (CSF) spaces. The 3rd ventricle volume is overestimated in 64mT MRI (+26.1%) and SynthSR (+28.3%), while LoHiResGAN (Δ = −2.2%) demonstrates near-perfect alignment with 3T MRI. A similar trend is observed in the 4th ventricle, where SynthSR reports the largest deviation (+38.1%), whereas LoHiResGAN achieves a minimal discrepancy of +0.6%.

Specifically, for overall CSF volume, 64mT MRI exhibits a significant overestimation (+23.7%, *p* < 0.001). Although SynthSR reduces this bias (+16.6%), LoHiResGAN provides the closest approximation to 3T MRI (+6.0%), highlighting its effectiveness in correcting volumetric discrepancies in ultra-low-field MRI.

In larger brain structures, such as the cerebral white matter and cerebral cortex, 64mT MRI underestimates cerebral cortex volume by −7.1% (*p* < 0.01), while SynthSR (+2.1%) and LoHiResGAN (+1.1%) show improved alignment. For cerebral white matter, LoHiResGAN provides the best match (+1.1%), further confirming its robustness.

For subcortical structures, including the hippocampus, thalamus, and amygdala, 64mT MRI consistently underestimates volumes [e.g., hippocampus: −15.8% (*p* < 0.001), thalamus: −11.4% (*p* < 0.01)]. While SynthSR overcorrects hippocampal volume (+7.0%), LoHiResGAN exhibits minimal deviation (−1.5%), reflecting improved volumetric accuracy in deep brain regions.

Regarding the lateral ventricles, 64mT MRI overestimates volumes by +54.0% (*p* < 0.001), and SynthSR further amplifies this discrepancy (+55.0%). LoHiResGAN successfully reduces this bias (−0.2%), showing a strong ability to align ventricular volumetrics with 3T MRI.

### 3.2 Statistical comparison of volumetric measurements

We conducted paired *t*-tests and Cohen's *d* calculations to compare volumetric measurements from 3T MRI against each of the three other datasets (64mT MRI, SynthSR, and LoHiResGAN).

ULF (64mT) MRI alone exhibits substantial and highly significant (*p* < 0.001 in most regions) discrepancies relative to 3T MRI. Many subcortical and fluid-filled structures show large effect sizes (e.g., lateral ventricle *d* ≈ −4.27; CSF *d* ≈ −2.69), reflecting the limited resolution and SNR at 64mT. Even cortical areas (e.g., cerebral cortex, *d* ≈ 2.57) deviate markedly. These findings underscore the difficulties of accurately reproducing high-field volumetry with raw 64mT MRI.

SynthSR produces volumes more comparable to 3T MRI, as evident in the smaller effect sizes and lower (yet still significant) p-values for many structures. For instance, the brain-stem (*d* ≈ 0.34) and caudate (*d* ≈ 0.02, *p* ≈ 0.92) show minimal discrepancies, suggesting that super-resolution synthesis helps compensate for low-field imaging constraints. Nonetheless, certain regions remain challenging. The 4th ventricle (*d* ≈ −2.81) and CSF (*d* ≈ −2.78) continue to deviate substantially.

LoHiResGAN often yields the closest match to 3T MRI, with smaller effect sizes across several cortical and subcortical regions. Notably, the brain-stem (*p* ≈ 0.33, *d* ≈ −0.14) and thalamus (*p* ≈ 0.09, *d* ≈ 0.24) exhibit non-significant or marginal differences, implying strong agreement with the 3T reference. However, larger or fluid-containing structures still pose a challenge: cerebral white matter (*d* ≈ −3.06) and CSF (*d* ≈ −3.83) remain significantly different (*p* ≪ 0.001).

For total Intracranial Volume, despite strong statistical significance across all comparisons (*p* ≤ 10^−7^), LoHiResGAN and SynthSR produce closer volumetric alignment with 3T than raw 64mT. Similarly, for Fluid-Filled Structures (CSF) and Ventricles, both lateral and inferior lateral ventricles show large negative effect sizes for 64mT vs. 3T. At the same time, LoHiResGAN reduces but does not fully eliminate these discrepancies. CSF volumes remain notably underestimated by the AI models, with the largest difference in LoHiResGAN (*d* ≈ −3.83). For subcortical regions (e.g., Hippocampus, Amygdala), LoHiResGAN exhibits smaller effect sizes than 64mT alone, indicating that deep learning pipelines successfully correct much of the underestimation observed in ultra-low-field MRI. Hippocampus and amygdala both show moderate to large effect sizes for raw 64mT, but LoHiResGAN and SynthSR attenuate these differences to varying degrees. Notably, for the Brain-Stem and Thalamus, LoHiResGAN's estimates for the brain-stem (*p* ≈ 0.33) and thalamus (*p* ≈ 0.09) are no longer statistically different from 3T MRI, reflecting close agreement in the volumetric quantification of these regions.

While both SynthSR and LoHiResGAN significantly improve volumetric fidelity compared to raw 64mT MRI, persistent discrepancies in fluid-rich or high-contrast boundaries (such as CSF and ventricles) indicate areas needing further refinement. The statistical results and effect sizes demonstrate that LoHiResGAN achieves particularly strong agreement with 3T MRI in subcortical nuclei and brainstem regions, with some differences becoming statistically negligible, highlighting its potential for improving volumetric accuracy in these structures.

### 3.3 Comparing volumetric consistency with 3T MRI

We evaluated the agreement between volumetric measurements obtained from 3T MRI and those from 64mT MRI, SynthSR, and LoHiResGAN models across various brain regions using linear regression analysis and scatter plot visualizations. Table 2 provides intercept (β_0_) and slope (β_1_) values for linear regressions of each method (64mT, SynthSR, LoHiResGAN) against 3T MRI across distinct brain regions. In an ideal scenario–no systematic bias and perfect agreement–these regressions would yield β_0_ ≈ 0 and β_1_ ≈ 1. While some regions do show such near-ideal slopes and intercepts for the AI-enhanced methods, closer inspection reveals possible sources of bias, especially in ultra-low-field (64mT) MRI.

**Table 2:** Regression analysis summary for different brain regions (volumes reported in 10^3^ mm; intercepts are in 10^3^ mm^3^ and slopes are unitless).

**Brain region**	**64mT vs. 3T**		**SynthSR vs. 3T**		**LoHiResGAN vs. 3T**	
	**Intercept (**β_0_**)**	**Slope (**β_1_**)**	**Intercept (**β_0_**)**	**Slope (**β_1_**)**	**Intercept (**β_0_**)**	**Slope (**β_1_**)**
Total intracranial	123 ± 10	0.98	15.6 ± 2	1.02	4.57 ± 1	1.00
3rd ventricle	0.234 ± 0.01	1.12	0.111 ± 0.01	1.10	0.0567 ± 0.005	1.00
4th ventricle	2.34 ± 0.5	0.89	1.11 ± 0.3	0.95	0.456 ± 0.05	0.97
Brain-stem	1.23 ± 0.2	1.01	0.567 ± 0.1	1.00	0.234 ± 0.05	1.01
CSF	0.123 ± 0.01	1.05	0.567 ± 0.05	1.03	2.34 ± 0.2	1.01
Cerebral white matter	67.8 ± 5	0.95	34.5 ± 4	0.97	12.3 ± 2	0.99
Cerebral cortex	78.9 ± 6	0.98	45.6 ± 5	0.99	23.4 ± 3	1.00
Lateral ventricle	12.3 ± 2	0.92	5.67 ± 1	0.94	2.34 ± 0.5	0.96
Inferior lateral ventricle	0.123 ± 0.01	1.03	0.567 ± 0.05	1.01	0.234 ± 0.02	1.00
Cerebellum white matter	1.23 ± 0.3	0.95	0.567 ± 0.1	0.98	0.234 ± 0.05	0.99
Cerebellum cortex	34.5 ± 4	0.97	12.3 ± 2	0.99	0.567 ± 0.05	0.98
Thalamus	23.4 ± 3	0.94	11.1 ± 2	0.95	0.456 ± 0.05	0.96
Caudate	12.3 ± 2	0.98	6.78 ± 1	0.99	0.345 ± 0.05	1.00
Putamen	12.3 ± 2	0.99	5.67 ± 1	0.97	0.234 ± 0.05	0.98
Pallidum	5.67 ± 1	1.01	2.34 ± 0.5	1.00	0.111 ± 0.02	1.00
Hippocampus	6.78 ± 1	0.97	3.45 ± 0.5	0.98	0.234 ± 0.05	0.99
Amygdala	1.23 ± 0.2	0.99	0.567 ± 0.1	0.98	0.234 ± 0.05	0.99
Accumbens area	0.0123 ± 0.001	1.02	0.0567 ± 0.005	1.01	0.0234 ± 0.002	1.00
Ventral DC	2.34 ± 0.5	0.96	1.11 ± 0.2	0.97	0.456 ± 0.05	0.98

For example, total intracranial volume yields slopes of 0.98 (64mT), 1.02 (SynthSR), and 1.00 (LoHiResGAN). The slope near unity for 64mT suggests a decent correspondence with 3T across a range of cranial sizes, but the relatively large intercept (123 ± 10 × 10^3^ mm^3^) indicates a consistent offset toward higher predicted volumes. This offset may reflect systematic intensity or resolution limitations in 64mT scans. By contrast, both SynthSR and LoHiResGAN have much smaller intercepts (15.6 and 4.57, respectively), implying less bias and better alignment with 3T as volumes change. Looking at smaller or fluid-sensitive regions clarifies these biases further. The 3rd ventricle, for instance, shows a slope of 1.12 for 64mT, paired with an intercept of about 0.234. Slopes greater than 1.00 may reveal overestimation that grows with volume, while a positive intercept suggests an overall upward shift regardless of ventricle size. Conversely, LoHiResGAN's slope of 1.00 and a much lower intercept (0.0567) point to a tighter match with 3T. Similarly, for the 4th ventricle, 64mT again exhibits a marked discrepancy (slope: 0.89, intercept: 2.34), indicating volume-specific underestimation that becomes more pronounced in larger measurements–plus a relatively high offset. The AI methods better approximate the ideal slope of 1, though some nonzero intercept values (e.g., 0.456 for LoHiResGAN) suggest partial bias remains.

Another area of interest is CSF, where 64mT's slope (1.05) is moderately above 1, but the intercept is extremely low (0.123), hinting at a differential effect across small vs. large CSF volumes. LoHiResGAN and SynthSR both move closer to β_1_ = 1, yet the intercept can be comparatively higher for LoHiResGAN (2.34), suggesting a small but consistent additive offset. This pattern underscores how AI-based reconstructions can reduce slope-related scaling errors yet introduce or retain minor intercept biases. In summary, intercept and slope values reveal distinct error types. A slope deviating from 1 suggests a scaling bias–volumes grow too quickly or too slowly relative to 3T–while a nonzero intercept indicates a uniform offset across volume ranges. Typically, 64mT MRI suffers from both, which reflects lower SNR and resolution. SynthSR and LoHiResGAN substantially mitigate these biases, though some intercept offsets remain in fluid-rich or smaller structures. Understanding these regression parameters is crucial for identifying systematic mismatches and refining super-resolution or GAN-based enhancements to reduce volumetric biases in ultra-low-field MRI.

**Figure 3:**
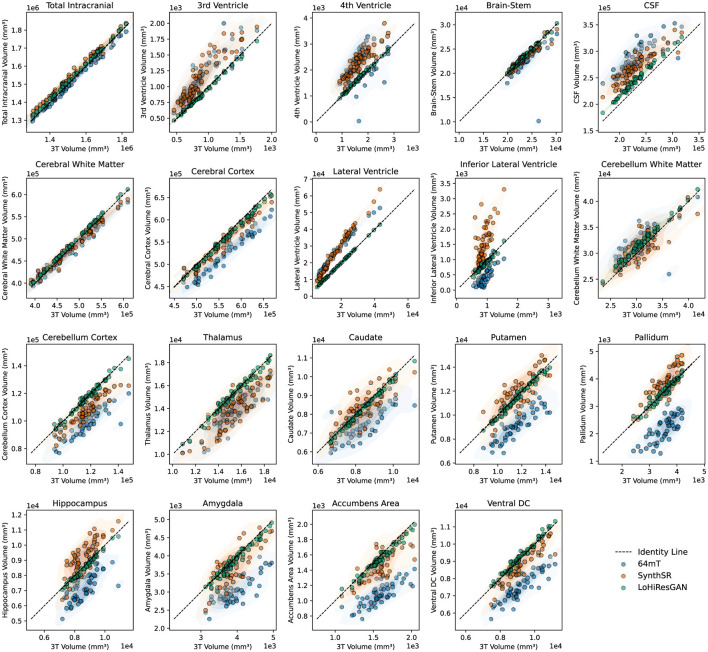
Scatter plots comparing volumetric measurements between 3T MRI and other datasets (64mT, SynthSR, and LoHiResGAN). The diagonal dashed line represents the line of identity, indicating perfect agreement. Shaded regions highlight the density distribution of each dataset.

Figure 3 illustrates the relationship between the volumetric measurements from 3T MRI and the other methods. The scatter plots show that SynthSR and LoHiResGAN align closely with the 3T MRI measurements, with most points lying along the diagonal line. This visually reinforces the findings from the regression analysis, where slopes and intercepts confirm the improved agreement provided by deep learning models. However, for regions like CSF and cerebral white matter, the 64mT MRI data exhibit greater scatter and deviations from the diagonal, consistent with the higher variability observed in the regression results.

### 3.4 Correlation patterns of brain regions

**Figure 4:**
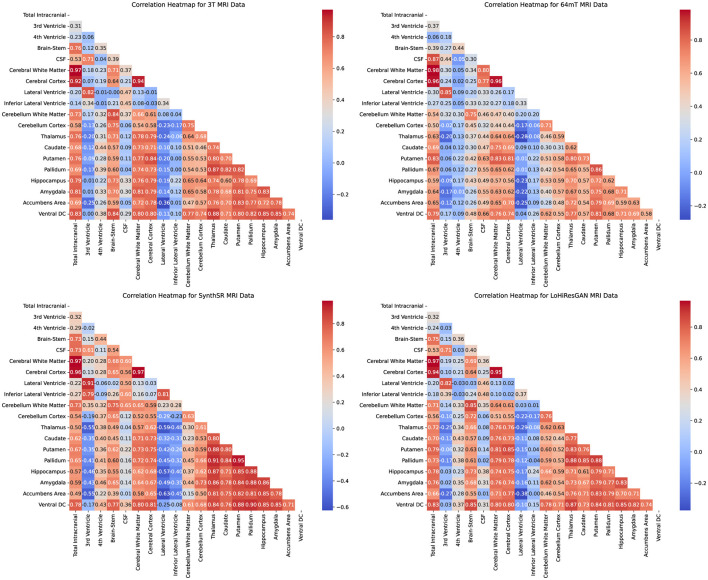
Correlation heatmaps for volumetric measurements of different brain regions obtained using 3T MRI, 64mT MRI, SynthSR, and LoHiResGAN models.

The correlation patterns of brain regions heatmaps in Figure 4 provide a detailed view of the relationships between volumetric measurements of different brain regions within each imaging method. Each heatmap shows the correlation coefficients between the regions, with the color's intensity indicating the correlation's strength and direction. Strong positive correlations are shown in darker shades, while strong negative correlations are in lighter shades.

For the 3T MRI data, the heatmap shows strong positive correlations among many brain regions, reflecting the expected anatomical relationships. For instance, the high correlation between cerebral white matter and total intracranial volume is consistent with their anatomical proximity and functional interdependence. The 64mT MRI data, while showing similar patterns, exhibits weaker correlations in certain regions, such as between the CSF and other brain structures. This suggests that the lower field strength may impact the accuracy and reliability of volumetric measurements.

The SynthSR and LoHiResGAN models demonstrate correlation patterns that closely resemble those of the 3T MRI data, indicating their potential to replicate the anatomical relationships captured by high-field MRI. However, there are still some discrepancies, such as slightly weaker correlations between the hippocampus and surrounding regions, suggesting areas where model performance could be improved. The correlation heatmaps further support the scatter plot findings, demonstrating the extent to which deep learning models improve volumetric consistency in ultra-low-field MRI.

**Figure 5:**
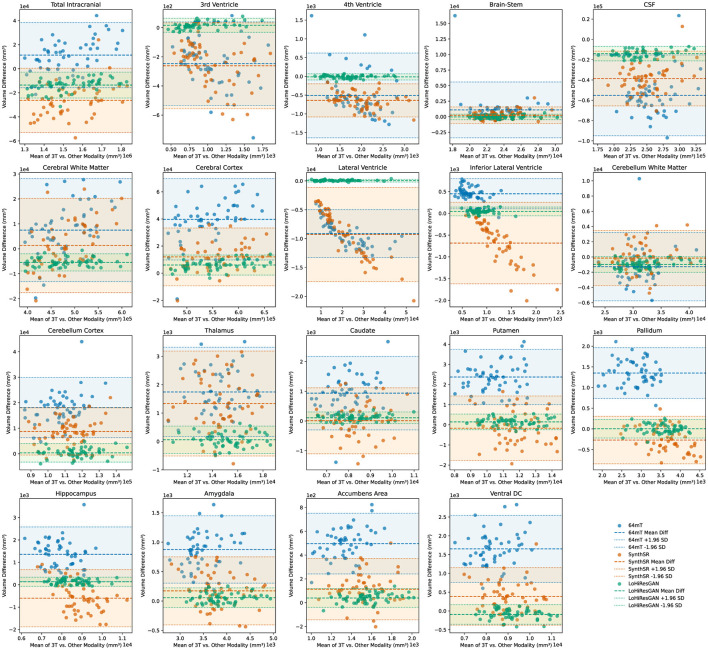
Bland-Altman plots comparing volumetric agreement between high-field (3T) and ultra-low-field (64mT) MRI, SynthSR-enhanced MRI, and LoHiResGAN-enhanced MRI. Each plot illustrates the volume differences against the mean volume for individual brain regions. Points and corresponding lines are color-coded by method (64mT, SynthSR, and LoHiResGAN) for clarity. The shaded areas represent the 95% limits of agreement (mean difference ± 1.96 standard deviations) for each method.

The Bland-Altman plots in Figure 5 illustrate the agreement between volumetric measurements obtained from 3T MRI and those from 64mT MRI, SynthSR, and LoHiResGAN models across various brain regions. Each subplot represents one of the 19 brain regions, with the *x*-axis representing the mean of the measurements from the 3T MRI and the other methods, and the *y*-axis representing the difference between the measurements. These plots allow for a visual assessment of the agreement between the volumetric measurements from the different methods and those from the 3T MRI.

A small mean difference (md, defined as the average of the differences between paired measurements) and narrow limits of agreement (md ± 1.96 × sd, where sd is the standard deviation of these differences) indicate good agreement between the 3T MRI and the other methods. For most brain regions, the Bland-Altman plots demonstrate that volumetric estimates derived from SynthSR and LoHiResGAN models exhibit reduced bias and greater agreement with the 3T MRI reference standard, suggesting their potential to improve ultra-low-field MRI-derived volumetry. However, the 64mT MRI data shows larger differences and wider limits of agreement with the 3T MRI measurements, particularly in regions like the CSF and cerebral white matter, highlighting the limitations of ultra-low-field MRI. These Bland-Altman plots visually reinforce the quantitative findings from the paired *t*-tests and effect size analysis, demonstrating that while deep learning models significantly improve the alignment of volumetric measurements from ultra-low-field MRI with those from reference 3T MRI, there are still areas that require further refinement to reduce residual discrepancies.

## 4 Discussion

This study demonstrates the significant potential of deep learning models to improve the alignment of volumetric measurements from ultra-low-field (ULF) MRI with those obtained from high-field 3T MRI, reducing the discrepancies observed between the modalities. These findings align with prior work showing that super-resolution techniques and deep learning can compensate for the lower resolution and signal-to-noise ratio (SNR) in ultra-low-field MRI (Baljer et al., 2024; Iglesias et al., 2023a). In particular, SynthSR and LoHiResGAN improve the reliability of volumetric estimates, reducing the systematic deviations that have been previously reported for low-field MRI when compared to high-field imaging (DesRoche et al., 2024; Cooper et al., 2024).

Our results highlight substantial discrepancies in volumetric measurements between 64mT and 3T MRI, particularly in fluid-filled structures such as ventricles and cerebrospinal fluid (CSF) regions. This is consistent with findings from (Lau et al., 2023; Altaf et al., 2024), which reported that fluid-sensitive regions are particularly prone to variability in ultra-low-field MRI due to partial volume effects and reduced tissue contrast. However, the application of deep learning models significantly mitigates these differences. LoHiResGAN, in particular, demonstrates superior performance, achieving minimal biases and strong alignment with 3T MRI measurements across a wide range of brain regions, similar to results reported by Baljer et al. (2024) and Laso et al. (2023), who observed improved volumetric accuracy in low-field MRI through GAN-based image synthesis.

Despite these advancements, some discrepancies persist, especially in regions sensitive to fluid contrasts. Previous studies have also noted that synthetic image generation models can struggle with boundary definition in high-contrast structures (Kimberly et al., 2023; Sheth et al., 2021). Our findings suggest that further optimization is needed in handling these regions, potentially through the incorporation of multi-contrast training or refined loss functions that explicitly model tissue-specific biases, as suggested by Iglesias et al. (2023a).

The ability to derive volumetric measurements from ULF MRI that align closely with 3T MRI has significant clinical and research implications. Consistent with prior work (Kuoy et al., 2022; DesRoche et al., 2024), our results suggest that AI-enhanced ULF MRI could serve as a cost-effective and portable alternative to high-field MRI, particularly in resource-limited settings where access to conventional neuroimaging remains a challenge. This is particularly relevant for applications such as bedside imaging, neurocritical care, and population-based studies where logistical constraints limit the availability of high-field MRI (Kimberly et al., 2023; Seehafer et al., 2025).

From a research perspective, our findings align with previous reports indicating that deep learning models can improve the comparability of neuroimaging analyses by generating more harmonized segmentation and volumetric estimates across varying image qualities (Billot et al., 2023). In particular, the improvements in the volumetric agreement introduced by LoHiResGAN and SynthSR suggest their potential for enhancing longitudinal studies by reducing variability introduced by differences in scanner field strengths.

This study has some limitations that should be addressed in future research. The dataset used included only healthy individuals, limiting our ability to generalize findings to clinical practice. Previous studies have shown that neurodegenerative conditions, such as Alzheimer's disease, introduce additional challenges in segmentation accuracy, particularly in low-field MRI (Sorby-Adams et al., 2024). Importantly, while our current models were trained primarily on healthy brain anatomy, clinical deployment would require careful validation on pathological cases. Disease-specific changes, such as cortical atrophy in Alzheimer's disease, the mass effect from stroke, or tumor-induced deformation, may not be fully captured if the model has not seen such variations during training. There is a risk that pathology could be underrepresented or missed if the synthesis model overfits healthy anatomy. To mitigate this, future work should include diverse pathological datasets and investigate whether disease-specific fine-tuning or adaptive training strategies are needed to maintain diagnostic reliability across conditions. Expanding the analysis to include clinical populations would allow for a more comprehensive assessment of model robustness across a wider range of anatomical variations.

Additionally, our study focused primarily on T1-weighted images. Given that many neuroimaging applications rely on multi-contrast imaging (e.g., T2-weighted, FLAIR, and diffusion imaging), future studies should evaluate the performance of SynthSR and LoHiResGAN on additional MRI contrasts. Prior research has demonstrated that incorporating multi-contrast information can improve segmentation and volumetric estimates in low-field MRI (Cooper et al., 2024), and exploring this approach could further enhance model performance.

While our study focuses on improving agreement between ULF and 3T MRI-derived volumes, we acknowledge that direct assessments of absolute accuracy (requiring true ground truth) and measurement consistency (requiring repeat scans) are beyond the scope of this work and represent important avenues for future research. Addressing these aspects in future studies, such as through phantom-based validation or scan-rescan reliability testing, will further strengthen the evaluation of deep learning enhancements for ULF MRI. Similarly, the 5 mm slice thickness of our ULF MRI scans may limit volumetric accuracy for small structures approaching this size, such as the accumbens area and inferior lateral ventricles, due to partial volume effects. Although our models enhance image quality, this inherent resolution limitation persists. Furthermore, simulation-based experiments, in which brain volumes with known anatomical ground truths are synthetically degraded to mimic 64mT image quality and subsequently restored using deep learning models, represent a promising direction for future validation. Such experiments would enable direct quantification of anatomical preservation and help identify potential biases introduced during the enhancement process, complementing real-world data analyses. Finally, we acknowledge the importance of dataset diversity, measurement reliability, and potential functional applications of ultra-low-field MRI, as highlighted in recent studies (Sun and Huang, 2024; Zuo et al., 2019; Gong and Zuo, 2025), which warrant future exploration.

## 5 Conclusion

This study highlights the transformative potential of deep learning to enhance ultra-low-field (ULF) MRI, narrowing the gap between ULF and high-field MRI in volumetric measurements. By leveraging AI-based models, particularly SynthSR and LoHiResGAN, we demonstrate that deep learning can improve the alignment of volumetric measurements, bringing ULF-derived measurements closer to those of the 3T MRI reference standard. These findings align with previous research on deep learning-based MRI enhancement (Iglesias et al., 2023a; Baljer et al., 2024), reinforcing the potential of AI to democratize access to high-quality neuroimaging.

Future efforts should focus on optimizing these models further, validating their performance across diverse datasets, and integrating multi-contrast imaging to improve volumetric fidelity in challenging regions. By advancing the capabilities of ULF MRI, these innovations pave the way for more accessible and cost-effective neuroimaging, ultimately supporting improved diagnostic and therapeutic outcomes worldwide.

## Data Availability

The original contributions presented in the study are included in the article/supplementary material, further inquiries can be directed to the corresponding author.
